# Supplementary figures and images for: Cortical beta oscillations reflect the contextual gating of visual action feedback
Source: Neuroimage. 2020 Nov 15;222:117267. doi: 10.1016/j.neuroimage.2020.117267 (PMC7779369; doi:10.1016/j.neuroimage.2020.117267)

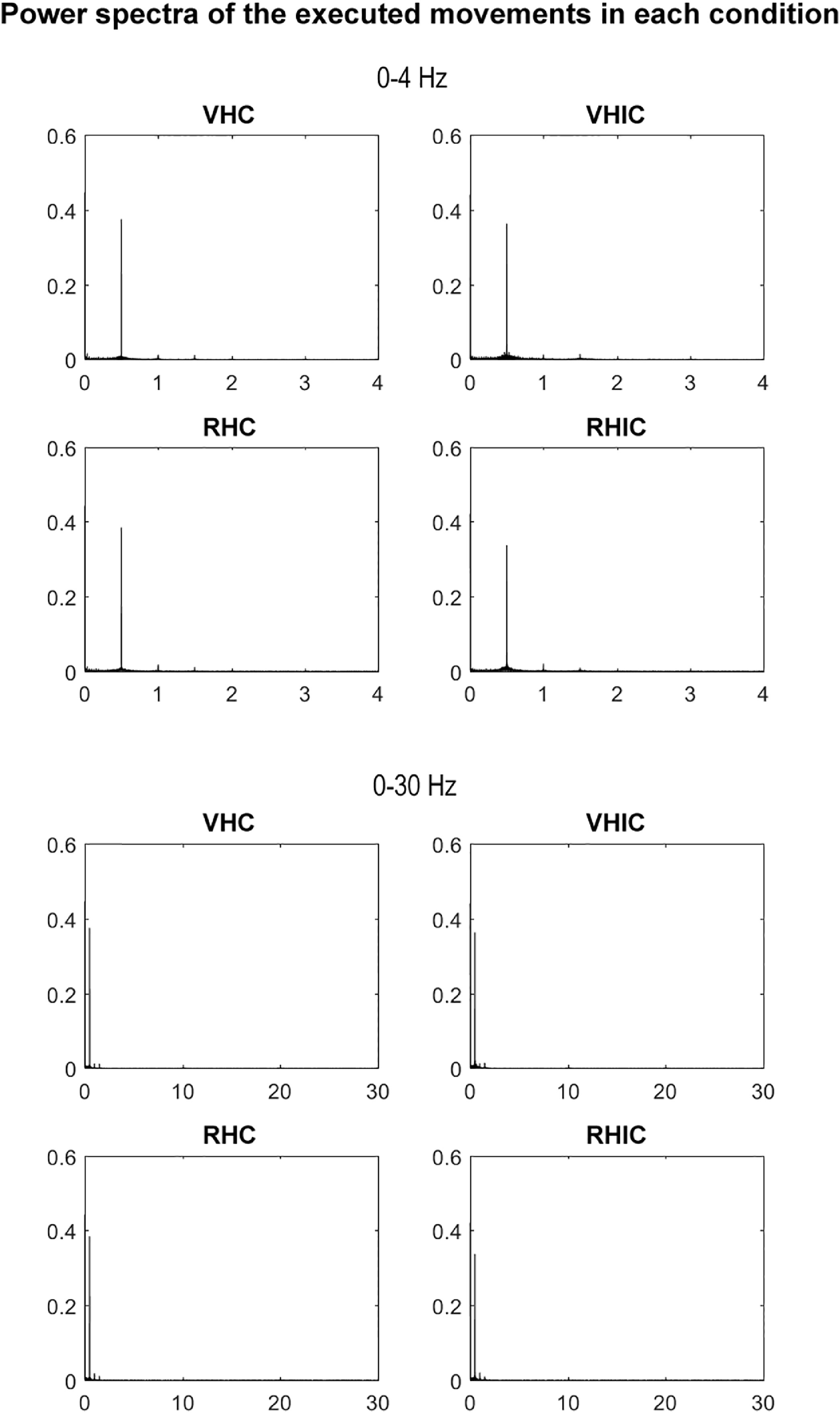

Supplement: Supplementary file 1 [file mmc1.jpg]
